# Incidence, characteristics and risk factors of hypofibrinogenemia associated with tigecycline: A multicenter retrospective study in China

**DOI:** 10.3389/fphar.2022.943674

**Published:** 2022-10-11

**Authors:** Bing Leng, Chengwu Shen, Tiantian Gao, Kai Zhao, Xuemei Zhao, Yujin Guo, Jiyong Wu, Jing Yang, Wei Fang, Jicheng Zhang, Yahui Zhang, Chao Sun, Lei Duan, Jing Huang, Yougang Qi, Genquan Yan

**Affiliations:** ^1^ Department of Pharmacy, Shandong Provincial Hospital Affiliated to Shandong First Medical University, Jinan, China; ^2^ Management Office of Information and Network, Shandong Provincial Hospital Affiliated to Shandong First Medical University, Jinan, China; ^3^ Department of Clinical Pharmacy, Jining No. 1 People’s Hospital, Jining, China; ^4^ Department of Pharmacy, Shandong Second Provincial General Hospital, Jinan, China; ^5^ Department of Pharmacy, Shandong Provincial Third Hospital, Jinan, China; ^6^ Department of Critical Care Medicine, Shandong Provincial Hospital Affiliated to Shandong First Medical University, Jinan, China; ^7^ Department of Clinical Pharmacy, The Second Hospital of Shandong University, Jinan, China; ^8^ Department of Pharmacy, Zibo Central Hospital, Zibo, China; ^9^ Department of Clinical Pharmacy, Honghui Hospital, Xi’an Jiaotong University, Xi’an, China; ^10^ Department of Pharmacy, The Second People’s Hospital of Liaocheng, Liaocheng, China

**Keywords:** tigecycline (TGC), adverse event (AE), hypofibrinogenemia, coagulation-flocculation, clinical characteristic, risk factor (RF)

## Abstract

**Background:** Tigecycline was recently found to cause coagulation disorders, especially hypofibrinogenemia, which may interfere with the administration of antimicrobial therapy. This study aimed to investigate the incidence and clinical characteristics of and risk factors for tigecycline-associated hypofibrinogenemia.

**Methods:** In this multicenter retrospective study, patients receiving tigecycline or imipenem–cilastatin to treat Gram-negative bacterial infections in nine Chinese tertiary hospitals between January 2020 and December 2020 were enrolled. Baseline data and coagulation variables were compared using cohort and case–control studies.

**Results:** Totals of 485 patients treated with tigecycline and 490 patients treated with imipenem–cilastatin were included in this study. Compared with imipenem–cilastatin, tigecycline was associated with reduced fibrinogen and prolonged activated partial thromboplastin time and prothrombin time (all *p* < 0.001), with the most remarkable change in fibrinogen (down by 48.0%). The incidence of hypofibrinogenemia in patients treated with tigecycline was >50%, with propensity score-matched analysis or not. The relative risk of hypofibrinogenemia with tigecycline versus imipenem–cilastatin was 2.947 (95% CI: 2.151–4.039) at baseline balance. Tigecycline-associated hypofibrinogenemia led to a higher incidence (12.1%) of bleeding events. However, none of supplemental therapies after withdrawal had an effect on the normalization of fibrinogen levels. The risk factors for tigecycline-associated hypofibrinogenemia were treatment duration ≥6 days (odds ratio [OR] 5.214, 95% confidence interval [CI] 2.957–9.191, *p* < 0.001), baseline fibrinogen <4 g/L (OR 4.625, 95% CI 2.911–7.346, *p* < 0.001), cumulative dose ≥1,000 mg (OR 2.637, 95% CI 1.439–4.832, *p* = 0.002), receiving CRRT (OR 2.436, 95% CI 1.179–5.031, *p* = 0.016), baseline PT > 14 s (OR 2.110, 95% CI 1.317–3.380, *p* = 0.002) and baseline total bilirubin >21 μmol/L (OR 1.867, 95% CI 1.107–3.147, *p* = 0.019), while the protective factor was skin and soft tissue infection (OR 0.110, 95% CI 0.026–0.473, *p* = 0.003).

**Conclusion:** The clinical characteristics of and risk factors for tigecycline-associated hypofibrinogenemia identified in this study can offer practical reference for the clinical management of patients.

## Introduction

Tigecycline is a first-in-class glycylcycline antibiotic with promising efficacy against a broad spectrum of microbes, including aerobic and facultative Gram-positive bacteria and Gram-negative bacteria (GNB) and anaerobic bacteria. It inhibits the synthesis of bacterial proteins at the level of the ribosome subunit (Qvist et al., 2012). Tigecycline was approved by the US Food and Drug Administration for the treatment of complicated intra-abdominal infections and complicated skin and skin-structure infections in 2005 and for community-acquired pneumonia in 2009. The authorized dose is 50 mg every 12 h following a 100 mg loading dose, which has proven to be effective and well tolerated. The most common adverse events recorded in the package insert are nausea and vomiting. Notably, tigecycline exhibited potent *in vitro* activity against multidrug-resistant (MDR) bacteria, such as extended-spectrum β-lactamase-producing *Enterobacteriaceae* and MDR *Acinetobacter baumannii*, due to its specific anti-resistance mechanism (Perez et al., 2007). Tigecycline is thus being increasingly prescribed for difficult-to-treat infections caused by MDR bacteria, especially MDR-GNB. However, off-label indications, high-dose regimens and long-term treatment have been frequently used in clinical practice, to ensure its effectiveness in severe infections caused by MDR bacteria (Leng et al., 2021), suggesting that the real-world adverse events of tigecycline might actually be more frequent and serious than those recorded in the package insert.

Tigecycline-associated hypofibrinogenemia was first reported in a case study by Pieringer et al. in 2010, which is currently a documented adverse event (Pieringer et al., 2010). A retrospective study in China found a decrease in fibrinogen in patients treated with tigecycline (Zhang et al., 2015) and several cases of potentially life-threatening coagulation disorders induced by tigecycline have also been reported (Akalay et al., 2021; Balfousias et al., 2019; Fan et al., 2020; Rossitto et al., 2014; Routsi et al., 2015; Sabanis et al., 2015; Wu et al., 2017; Yılmaz Duran et al., 2018). However, although various reports have demonstrated an association between tigecycline and hypofibrinogenemia, the findings could not provide practical reference for clinical administration of tigecycline due to limitations such as small sample sizes, lack of a control group and insufficient description. We therefore conducted multicenter retrospective cohort and case-control studies to compare the coagulation parameters between patients treated with tigecycline and imipenem–cilastatin and to investigate the underlying characteristics of and risk factors for tigecycline-associated hypofibrinogenemia.

## Materials and methods

### Study design and patient selection

This was a multimethod, multicenter, retrospective investigation of hospitalized Chinese patients receiving tigecycline/imipenem–cilastatin at nine hospitals, including a cohort study using propensity score-matched analysis and a case-control study. All participating centers (viz., Shandong Provincial Hospital Affiliated to Shandong First Medical University, The Second Hospital of Shandong University, Shandong Provincial Third Hospital, Shandong Second Provincial General Hospital, Jining No. 1 People’s Hospital, Xi’an Honghui Hospital, Zibo Central Hospital, The Second People’s Hospital of Liaocheng and Qufu City People’s Hospital) were tertiary care hospitals capable of diagnosing and treating infectious diseases. Ethical approval was granted by the medical ethics committee of Shandong Provincial Hospital Affiliated to Shandong First Medical University and the participating hospitals.

All hospitalized patients with definite or suspected GNB infection who were administered either tigecycline or imipenem–cilastatin between January 2020 and December 2020 at any of the nine included hospitals were eligible for inclusion. The exclusion criteria were: 1) treatment duration <3 days; 2) congenital and acquired coagulation disorders with prolongation of activated partial thromboplastin time (aPTT) > 10 s, prolongation of prothrombin time (PT) > 3 s or fibrinogen <2 g/L prior to initiation; and 3) lack of clinical data or laboratory data before and during administration in medical records.

### Cohort study and propensity score matching

In this retrospective cohort study, we collected basic data for the enrolled patients and compared the baseline characteristics and coagulation data between patients treated with tigecycline and patients treated with imipenem–cilastatin. There were significant differences in some baseline characteristics between the two cohorts, propensity score matching was therefore applied to balance these variables.

### Case-control study

To explore the risk factors for tigecycline-associated hypofibrinogenemia, a case-control study was used to analyze data from all patients treated with tigecycline. Treating the development of hypofibrinogenemia as an outcome, patients receiving tigecycline were divided into a hypofibrinogenemia and a non-hypofibrinogenemia group. We then assessed the relationship between tigecycline-associated hypofibrinogenemia and patient demographics, comorbidities, medication regimens, combination therapies, important invasive procedures and baseline laboratory tests, based on clinical knowledge of potential risk factors.

### Data collection

In this retrospective analysis, data pertaining to patient demographics (age and sex), ward, comorbidities (diabetes mellitus, hypertension, chronic heart failure, atrial fibrillation, chronic obstructive pulmonary disease, chronic kidney disease, chronic liver disease, malignancy and shock), microbiological data, prescription of tigecycline and imipenem–cilastatin, combination therapies (cefoperazone–sulbactam, sodium valproate, asparaginase, vasopressors, anticoagulants and anti-platelet aggregation drugs), invasive procedures (mechanical ventilation and continuous renal replacement therapy [CRRT]), bleeding events and laboratory data (fibrinogen, aPTT, PT, alanine aminotransferase, aspartate aminotransferase, total bilirubin, albumin and serum creatinine) were acquired from patient medical records. Hypofibrinogenemia was defined as a fibrinogen level <2.0 g/L. Alanine aminotransferase or aspartate aminotransferase >120 U/L and total bilirubin >21 μmol/L were considered to be elevated. Serum albumin <30 g/L was considered to be reduced. Serum creatinine >135 μmol/L in males or >105 μmol/L in females was considered to be elevated.

### Statistical analysis

Data are presented as rates for categorical variables and medians (interquartile range [IQR]) for continuous variables. Categorical variables were analyzed with χ^2^ or Fisher’s exact tests and continuous data were evaluated using Mann–Whitney U tests. In the cohort study, propensity score matching was used to adjust for significant differences in baseline characteristics. In the case-control study, baseline characteristics of patients with and without hypofibrinogenemia were compared using χ^2^/Fisher’s exact tests or Mann-Whitney U tests for univariate analysis. Variables that were statistically significant in univariate analyses were entered into a multivariate logistic regression model to identify risk factors for tigecycline-associated hypofibrinogenemia, with odds ratios (ORs) and 95% confidence intervals (CIs). Correlations between coagulation parameters were identified by Pearson’s correlation analysis where appropriate. A two-tailed *p* value < 0.05 was considered significant for all studies.

## Results

### Demographics and clinical characteristics

**FIGURE 1:**
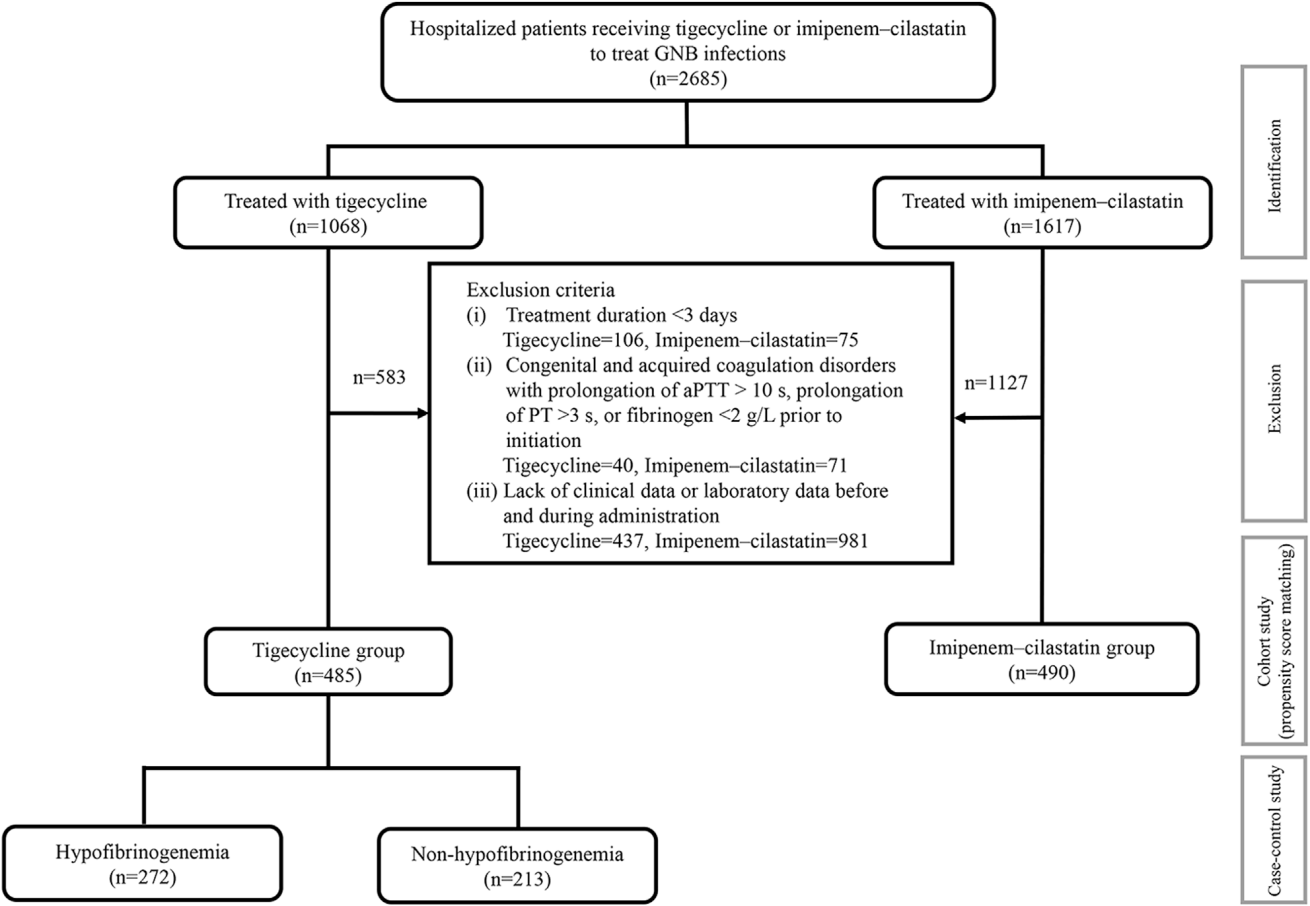
The detailed study process. 975 patients were enrolled from 2,685 hospitalized patients with definite or suspected infections of GNB into this study. A cohort study was performed in 485 patients receiving tigecycline and 490 patients receiving imipenem-cilastatin. Propensity score-matched analysis was used for balancing baseline characteristics. 272 patients with hypofibrinogenemia in the tigecycline treatment were compared with 213 non-hypofibrinogenemia patients in the same cohort using the case-control study. aPTT: activated partial thromboplastin time; PT: prothrombin time.

In total, 2,685 inpatients with definite or suspected infections caused by GNB in medical or surgical wards or intensive care units (ICUs) were screened and 975 patients were ultimately enrolled, including 485 patients treated with tigecycline and 490 patients treated with imipenem–cilastatin (Figure 1). The demographics and clinical characteristics of these patients are summarized in Table 1. The patient median age was 61.0 years and 32.0% were female. The percentages of ICU admissions were similar in the tigecycline and imipenem–cilastatin groups. Diabetes mellitus, hypertension, chronic heart failure and atrial fibrillation were significantly more prevalent in the tigecycline group, while there were no significant differences in the incidences of other comorbidities between the two cohorts. The types of infection mainly included pneumonia, intra-abdominal infection, bloodstream infection and skin and soft tissue infection. Patients treated with tigecycline were more likely to receive mechanical ventilation (*p* < 0.001) and CRRT (*p* = 0.047) than patients treated with imipenem–cilastatin. The median treatment durations of tigecycline and imipenem–cilastatin were 11.0 and 8.0 days (*p* < 0.001), respectively.

**TABLE 1:** Patient characteristics.

Variable	Total (*n* = 975)	Tigecycline (*n* = 485)	Imipenem–cilastatin (*n* = 490)	*p* value
Age (years)	61.0 (42.0–71.0)	62.0 (45.0–73.0)	60.0 (38.0–70.0)	0.001
Sex, female (%)	312 (32.0%)	139 (28.7%)	173 (35.3%)	0.026
ICU admission (%)	448 (45.9%)	219 (45.2%)	229 (46.7%)	0.621
Comorbidities (%)				
Diabetes mellitus	158 (16.2%)	91 (18.8%)	67 (13.7%)	0.031
Hypertension	285 (29.2%)	170 (35.1%)	115 (23.5%)	<0.001
Chronic heart failure	63 (6.5%)	46 (9.5%)	17 (3.5%)	<0.001
Atrial fibrillation	47 (4.8%)	42 (8.7%)	5 (1.0%)	<0.001
Chronic obstructive pulmonary disease	30 (3.1%)	20 (4.1%)	10 (2.0%)	0.060
Chronic kidney disease	127 (13.0%)	68 (14.0%)	59 (12.0%)	0.358
Chronic liver disease	150 (15.4%)	76 (15.7%)	74 (15.1%)	0.806
Malignancy	365 (37.4%)	171 (35.3%)	194 (39.6%)	0.162
Shock	280 (28.7%)	146 (30.1%)	134 (27.3%)	0.342
Type of infection (%)				<0.001
Pneumonia	573 (58.8%)	339 (69.9%)	234 (47.8%)	
Intra-abdominal infection	262 (26.9%)	81 (16.7%)	181 (36.9%)	
Bloodstream infection	90 (9.2%)	43 (8.9%)	47 (9.6%)	
Skin and soft tissue infection	33 (3.4%)	15 (3.1%)	18 (3.7%)	
Others	17 (1.7%)	7 (1.4%)	10 (2.0%)	
Incidence of GNB isolated (%)	521 (53.4%)	317 (65.4%)	204 (41.6%)	<0.001
Mechanical ventilation (%)	345 (35.4%)	198 (40.8%)	147 (30.0%)	<0.001
CRRT (%)	109 (11.2%)	64 (13.2%)	45 (9.2%)	0.047
Duration of administering antibiotics (days)	9.0 (7.0–14.0)	11.0 (8.0–15.0)	8.0 (6.0–12.0)	<0.001

Data are expressed as medians (interquartile range), or number (percentage). ICU: intensive care unit; GNB: Gram-negative bacteria; CRRT: continuous renal replacement therapy.

The results of coagulation tests are described in Table 2. There was no significant difference in baseline fibrinogen level or PT (*p* = 0.978 and 0.339, respectively) between the tigecycline and imipenem–cilastatin groups, but baseline aPTT was significantly lower in the tigecycline group (*p* = 0.043). After administration of the antibiotics, fibrinogen was significantly lower in the tigecycline group compared with the imipenem–cilastatin group (*p* < 0.001), while aPTT and PT were both significantly higher in the tigecycline group (both *p* < 0.001). The incidence of hypofibrinogenemia after treatment was significantly higher in the tigecycline group compared with the imipenem–cilastatin group (56.1% vs. 12.4%, *p* < 0.001).

**TABLE 2:** Comparison of coagulation tests between tigecycline and imipenem–cilastatin groups in the original and matched data sets.

Variable	Original data set			Matched data set		
	Tigecycline	Imipenem–cilastatin	*p* value	Tigecycline	Imipenem–cilastatin	*p* value
	(*n* = 485)	(*n* = 490)		(*n* = 213)	(*n* = 213)	
Baseline						
Fibrinogen (g/L)	3.98 (3.10–4.81)	3.92 (2.94–5.02)	0.978	3.95 (3.09–4.92)	3.99 (3.00–5.08)	0.934
aPTT (s)	31.80 (28.50–37.80)	32.85 (29.50–38.40)	0.043	32.30 (28.80–39.45)	32.90 (29.35–38.25)	0.814
PT (s)	14.00 (12.80–15.50)	14.10 (12.60–16.00)	0.339	14.10 (13.00–15.65)	14.20 (12.60–16.20)	0.796
After administration						
Fibrinogen (g/L)	1.84 (1.29–2.61)	3.30 (2.49–4.31)	<0.001	1.92 (1.31–2.60)	3.20 (2.28–4.28)	<0.001
aPTT (s)	42.50 (35.30–53.50)	38.15 (31.20–46.90)	<0.001	42.55 (34.55–55.83)	38.30 (31.70–47.75)	<0.001
PT (s)	16.90 (15.00–19.80)	15.20 (13.70–17.40)	<0.001	17.30 (15.00–19.85)	15.20 (13.70–17.50)	<0.001

Data are expressed as medians (interquartile range). aPTT: activated partial thromboplastin time; PT: prothrombin time.

### Propensity score-matched analysis

The baseline characteristics of patients who received tigecycline or imipenem–cilastatin (Table 1) were matched based on propensity scores to create two cohorts of 213 patients each and the balance of covariates was substantially improved between the matched cohorts (Table 3). There was no significant difference in baseline fibrinogen, aPTT, or PT (*p* = 0.934, 0.814 and 0.796, respectively) between the two matched antibiotic cohorts. However, after antibiotic administration, fibrinogen levels were significantly lower while aPTT and PT values were significantly higher in the tigecycline group compared with the imipenem–cilastatin group (all *p* < 0.001), in line with the results prior to propensity matching (Table 2). The incidence of hypofibrinogenemia remained significantly higher in the tigecycline group compared with the imipenem–cilastatin group after propensity matching (52.6% vs. 17.8%, *p* < 0.001). The relative risk of hypofibrinogenemia with tigecycline versus imipenem–cilastatin was 2.947 (95% CI: 2.151–4.039).

**TABLE 3:** Patient characteristics after 1:1 propensity score matching.

Variable	Total	Tigecycline	Imipenem–cilastatin	*p* value
	(*n* = 426)	(*n* = 213)	(*n* = 213)	
Age (years)	58.5 (39.0–69.0)	54.0 (37.0–69.0)	61.0 (45.0–70.0)	0.196
Sex, female (%)	134 (31.5%)	70 (32.9%)	64 (30.0%)	0.531
ICU admission (%)	200 (46.9%)	96 (45.1%)	104 (48.8%)	0.437
Comorbidities (%)				
Diabetes mellitus	55 (12.9%)	32 (15.0%)	23 (10.8%)	0.193
Hypertension	108 (25.4%)	60 (28.2%)	48 (22.5%)	0.181
Chronic heart failure	16 (3.8%)	9 (4.2%)	7 (3.3%)	0.610
Atrial fibrillation	3 (0.7%)	0 (0.0%)	3 (1.4%)	0.248
Chronic obstructive pulmonary disease	9 (2.1%)	4 (1.9%)	5 (2.3%)	1.000
Chronic kidney disease	49 (11.5%)	27 (12.7%)	22 (10.3%)	0.448
Chronic liver disease	66 (15.5%)	34 (16.0%)	32 (15.0%)	0.789
Malignancy	170 (39.9%)	86 (40.4%)	84 (39.4%)	0.843
Shock	123 (28.9%)	62 (29.1%)	61 (28.6%)	0.915
Type of infection (%)				0.072
Pneumonia	247 (58.0%)	126 (59.2%)	121 (56.8%)	
Intra-abdominal infection	109 (25.6%)	45 (21.1%)	64 (30.0%)	
Bloodstream infection	48 (11.3%)	26 (12.2%)	22 (10.3%)	
Skin and soft tissue infection	16 (3.8%)	11 (5.2%)	5 (2.3%)	
Others	6 (1.4%)	5 (2.3%)	1 (0.5%)	
Incidence of GNB isolated (%)	234 (54.9%)	115 (54.0%)	119 (55.9%)	0.697
Mechanical ventilation (%)	155 (36.4%)	78 (36.6%)	77 (36.2%)	0.920
CRRT (%)	54 (12.7%)	26 (12.2%)	28 (13.1%)	0.771
Duration of administering antibiotics (days)	9.0 (7.0–14.0)	9.0 (8.0–13.0)	9.0 (6.0–14.0)	0.356

Data are expressed as medians (interquartile range), or number (percentage).

### Effects of tigecycline treatment on coagulation function

**FIGURE 2:**
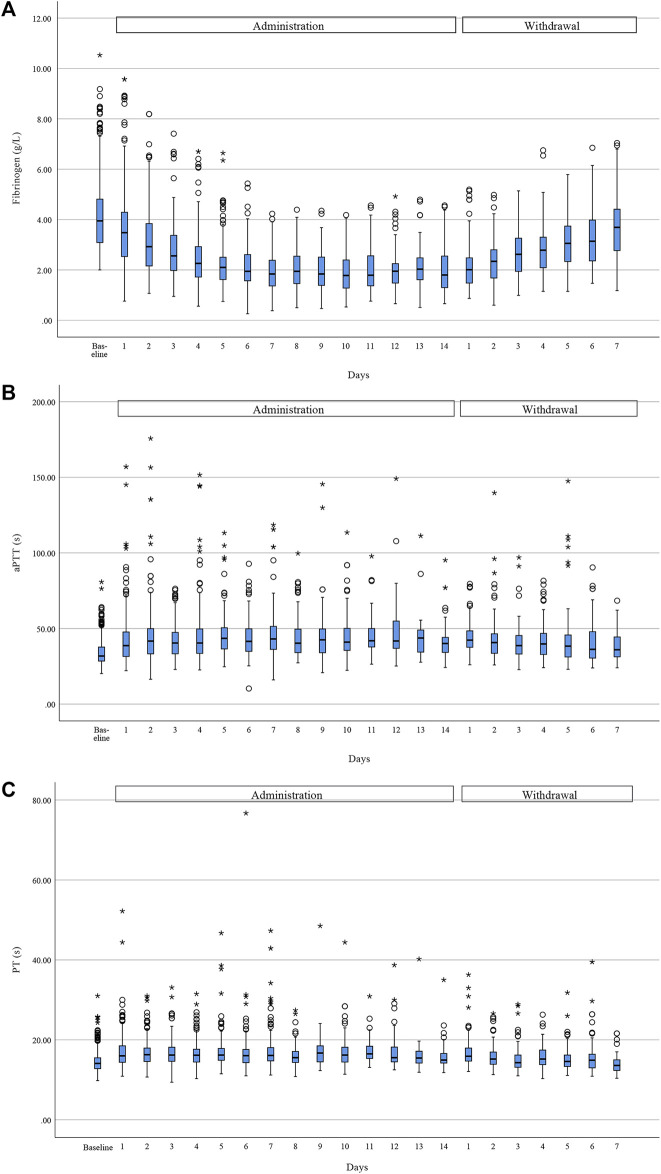
Trend of coagulation function during tigecycline administration and after withdrawal. Fibrinogen **(A)** showed a downward trend during administration and a rebound after withdrawal, while aPTT **(B)** and PT **(C)** rose during treatment and fell after withdrawal. Horizontal bars represent the median value, boxes represent the interquartile range, whiskers represent the 5th and 95th percentiles and hollow circle and asterisk indicate the outlier. (Due to the different course of administration and the relatively few data at the late stage of administration and withdrawal, we only showed trend of coagulation for the first 14 days of administration period and the first 7 days after withdrawal).

Given that tigecycline had a greater effect on coagulation than imipenem–cilastatin, we assessed the coagulation trends in 485 patients receiving tigecycline. The median of change in fibrinogen, aPTT and PT was 1.91 g/L (48.0%), 9.20 s (28.9%), and 2.80 s (20.0%), respectively (Figure 2). Pearson’s correlation analysis showed a weak but significant correlation between aPTT prolongation and PT prolongation (*r* = 0.604, *p* < 0.001), but no correlation between fibrinogen reduction and aPTT/PT prolongation (*r* = −0.096, *p* < 0.001 and *r* = −0.050, *p* = 0.273, respectively).

A total of 272 (56.1%) patients treated with tigecycline met the criteria for hypofibrinogenemia, which usually occurred a median of 6.0 (3.0–8.0) days after the initiation of administration. 219 (80.5%) of these patients had fibrinogen levels of 1.0–2.0 g/L and 53 (19.5%) had fibrinogen levels <1.0 g/L. Compared with patients without hypofibrinogenemia, patients with hypofibrinogenemia had a higher incidence of new bleeding events during treatment (8/213 [3.8%] vs. 33/272 [12.1%], *p* = 0.001), mainly gastrointestinal hemorrhage (19/33 [57.6%]) or mucocutaneous hemorrhage (7/33 [21.2%]). 44 patients with hypofibrinogenemia who received fibrinogen concentrate during tigecycline administration, only 10 (22.7%) patients of them returned to normal levels of fibrinogen.

A total of 108 patients with hypofibrinogenemia in this study were fully followed-up for coagulation parameters after withdrawal of the drug, with a median time to normalize fibrinogen levels of 4.0 (3.0–6.0) days. However, the other patients with hypofibrinogenemia were discharged, died, or had no coagulation parameters measured after drug discontinuation. Fifty of the followed-up patients (46.3%) received supplemental therapy after discontinuation, but their recovery times were similar to the 58 (53.7%) patients without supplementation (3.5 [3.0–5.0] vs. 4.5 [2.8–7.3] days, *p* = 0.211). The three commonly used supplemental therapies were vitamin K1, plasma/cryoprecipitate and fibrinogen concentrate, with no significant difference in recovery times of fibrinogen with or without these supplements (vitamin K1: 4.0 [3.0–5.0] vs. 3.0 [2.0–6.0] days, *p* = 0.671; plasma/cryoprecipitate: 3.0 [2.0–5.3] vs. 4.0 [3.0–5.0] days, *p* = 0.342; fibrinogen concentrate: 4.0 [2.0–5.0] vs. 3.0 [3.0–5.5] days, *p* = 0.835).

### Risk factors for tigecycline-associated hypofibrinogenemia

We compared the 272 patients with hypofibrinogenemia in the tigecycline group with 213 patients without hypofibrinogenemia in the same group using a case-control study design. The characteristics of the patients are listed in Table 4. There were no significant differences between the two groups in terms of demographics or baseline comorbidities. However, there were significant differences in the proportions of intra-abdominal infection and skin and soft tissue infection between the two groups (19.9% vs. 12.7%, *p* = 0.038; 1.1% vs. 5.6%, *p* = 0.006). Bleeding events before treatment were significantly more prevalent in patients with hypofibrinogenemia than in those without hypofibrinogenemia (12.1% vs. 5.6%, *p* = 0.014). Patients with hypofibrinogenemia had higher rates of baseline fibrinogen <4 g/L (64.0% vs. 33.8%, *p* < 0.001), baseline PT > 14 s (56.3% vs. 40.4%, *p* = 0.001) and baseline total bilirubin >21 μmol/L (38.9% vs. 22.4%, *p* < 0.001) compared with patients without hypofibrinogenemia, but there were no significant differences in other baseline laboratory tests.

**TABLE 4:** Comparison of demographic and clinical characteristics of patients with hypofibrinogenemia and non-hypofibrinogenemia during tigecycline administration.

Variables	Total of patients (*n* = 485)	Hypofibrinogenemia (*n* = 272)	Non-hypofibrinogenemia (*n* = 213)	Univariate analysis	Multivariate logistic regression analysis	
				*p* value	OR (95%CI)	*p* value
Age (years)	62.0 (45.0–73.0)	62.0 (44.0–74.0)	62.0 (46.0–72.5)	0.866		
Sex, female (%)	139 (28.7%)	87 (32.0%)	52 (24.4%)	0.067		
ICU admission (%)	219 (45.2%)	126 (46.3%)	93 (43.7%)	0.559		
Comorbidities (%)						
Diabetes mellitus	91 (18.8%)	49 (18.0%)	42 (19.7%)	0.633		
Hypertension	170 (35.1%)	90 (33.1%)	80 (37.6%)	0.306		
Chronic heart failure	46 (9.5%)	27 (9.9%)	19 (8.9%)	0.707		
Atrial fibrillation	42 (8.7%)	29 (10.7%)	13 (6.1%)	0.076		
Chronic obstructive pulmonary disease	20 (4.1%)	12 (4.4%)	8 (3.8%)	0.718		
Chronic kidney disease	68 (14.0%)	40 (14.7%)	28 (13.1%)	0.623		
Chronic liver disease	76 (15.7%)	44 (16.2%)	32 (15.0%)	0.729		
Malignancy	171 (35.3%)	105 (38.6%)	66 (31.0%)	0.081		
Shock	146 (30.1%)	86 (31.6%)	60 (28.2%)	0.411		
Type of infection (%)						
Pneumonia	339 (69.9%)	190 (69.9%)	149 (70.0%)	0.981		
Intra-abdominal infection	81 (16.7%)	54 (19.9%)	27 (12.7%)	0.038	1.377 (0.714–2.655)	0.339
Bloodstream infection	43 (8.9%)	23 (8.5%)	20 (9.4%)	0.720		
Skin and soft tissue infection	15 (3.1%)	3 (1.1%)	12 (5.6%)	0.006	0.110 (0.026–0.473)	0.003
Others	7 (1.4%)	2 (0.7%)	5 (2.3%)	0.249		
Bleeding event before treatment (%)	45 (9.3%)	33 (12.1%)	12 (5.6%)	0.014	2.300 (0.964–5.489)	0.061
Baseline laboratory tests (%)						
Fibrinogen<4 g/L	246 (50.7%)	174 (64.0%)	72 (33.8%)	<0.001	4.625 (2.911–7.346)	<0.001
aPTT >34 s	181 (37.3%)	103 (37.9%)	78 (36.6%)	0.778		
PT > 14 s	239 (49.3%)	153 (56.3%)	86 (40.4%)	0.001	2.110 (1.317–3.380)	0.002
Alanine aminotransferase >120U/L	33 (6.8%)	17 (6.3%)	16 (7.5%)	0.582		
Aspartate aminotransferase >120U/L	38 (7.9%)	24 (9.0%)	14 (6.7%)	0.359		
Total bilirubin >21 μmol/L	152 (31.7%)	105 (38.9%)	47 (22.4%)	<0.001	1.867 (1.107–3.147)	0.019
Albumin <30 g/L	160 (33.4%)	98 (36.4%)	62 (29.5%)	0.112		
Serum creatinine >135 μmol/L (male)/>105 μmol/L (female)	66 (13.8%)	42 (15.6%)	24 (11.4%)	0.187		
Therapeutic regimen (%)						
First dose				0.997		
50 mg	71 (14.6%)	40 (14.7%)	31 (14.6%)			
100 mg	384 (79.2%)	215 (79.0%)	169 (79.3%)			
200 mg	28 (5.8%)	16 (5.9%)	12 (5.6%)			
<50 mg^a^	2 (0.4%)	1 (0.4%)	1 (0.5%)			
Maintenance dose				0.028	1.268 (0.866–1.856)	0.223
50 mg q12h	196 (40.4%)	95 (34.9%)	101 (47.4%)			
100 mg q12h	239 (49.3%)	143 (52.6%)	96 (45.1%)			
50 mg q12h/100 mg q12h^b^	48 (9.9%)	33 (12.1%)	15 (7.0%)			
<50 mg q12h^a^	2 (0.4%)	1 (0.4%)	1 (0.5%)			
Treatment duration ≥6 days^c^	351 (72.6%)	238 (87.5%)	113 (53.1%)	<0.001	5.214 (2.957–9.191)	<0.001
Cumulative dose ≥1000 mg^d^	339 (69.9%)	225 (82.7%)	114 (53.5%)	<0.001	2.637 (1.439–4.832)	0.002
Combination therapies (%)						
Cefoperazone-sulbactam	189 (39.0%)	109 (40.1%)	80 (37.6%)	0.573		
Asparaginase/l-Asparaginase	6 (1.2%)	3 (1.1%)	3 (1.4%)	1.000		
Sodium valproate	14 (2.9%)	6 (2.2%)	8 (3.8%)	0.312		
Vasopressor	139 (28.7%)	78 (28.7%)	61 (28.6%)	0.993		
Norepinephrine	113 (23.3%)	64 (23.5%)	49 (23.0%)	0.892		
Dopamine	43 (8.9%)	27 (9.9%)	16 (7.5%)	0.353		
Others	37 (7.6%)	18 (6.6%)	19 (8.9%)	0.343		
Anticoagulant	206 (42.5%)	113 (41.5%)	93 (43.7%)	0.640		
Heparin	99 (20.4%)	58 (21.3%)	41 (19.2%)	0.574		
Low-molecular-weight heparin	125 (25.8%)	65 (23.9%)	60 (28.2%)	0.286		
Warfarin	2 (0.4%)	1 (0.4%)	1 (0.5%)	1.000		
Others	10 (2.1%)	5 (1.8%)	5 (2.3%)	0.755		
Anti-platelet aggregation drugs	31 (6.4%)	13 (4.8%)	18 (8.5%)	0.101		
Aspirin	17 (3.5%)	8 (2.9%)	9 (4.2%)	0.445		
Clopidogrel	17 (3.5%)	7 (2.6%)	10 (4.7%)	0.207		
Mechanical ventilation	198 (40.8%)	115 (42.3%)	83 (39.0%)	0.461		
CRRT	64 (13.2%)	47 (17.3%)	17 (8.0%)	0.003	2.436 (1.179–5.031)	0.016

Data are expressed as medians (interquartile range), or number (percentage). OR: odds ratio; 95% CI: 95%confidence interval.

a Patients received <50 mg of tigecycline for their small body weight.

b There was a dose change between 50 mg q12h and 100 mg q12h during the treatment.

c The period of tigecycline administration during coagulation monitoring was considered as treatment duration.

d Cumulative dose was based on treatment duration during coagulation monitoring.

Therapeutic strategies involving treatment duration ≥6 days (87.5% vs. 53.1%, *p* < 0.001) and cumulative dose ≥1,000 mg (82.7% vs. 53.5%, *p* < 0.001) were more likely to cause hypofibrinogenemia. Notably, to evaluate the effect of treatment duration on tigecycline-associated hypofibrinogenemia accurately, we considered the period of tigecycline administration during coagulation monitoring as treatment duration (median 8.0 days; 10.0 days in patients with hypofibrinogenemia vs. 6.0 days in patients without hypofibrinogenemia, *p* < 0.001). Maintenance dosage regimens of tigecycline were significantly different between the hypofibrinogenemia and non-hypofibrinogenemia groups (*p* = 0.028), whereas the first dosage regimens were similar (*p* = 0.997). Combined drugs, including cefoperazone–sulbactam, asparaginase, sodium valproate, vasopressors, anticoagulants and anti-platelet aggregation drugs, were used in similar proportions in both groups of patients. In addition, CRRT (17.3% vs. 8.0%, *p* = 0.003) rather than mechanical ventilation (*p* = 0.461) significantly affected the occurrence of hypofibrinogenemia.

All the rational variables that were significant at *p* < 0.05 in univariate analyses were incorporated into multivariate analysis. Ultimately, multiple logistic regression analysis indicated that treatment duration ≥6 days (OR 5.214, 95% CI 2.957–9.191, *p* < 0.001), baseline fibrinogen <4 g/L (OR 4.625, 95% CI 2.911–7.346, *p* < 0.001), cumulative dose ≥1,000 mg (OR 2.637, 95% CI 1.439–4.832, *p* = 0.002), receiving CRRT (OR 2.436, 95% CI 1.179–5.031, *p* = 0.016), baseline PT > 14 s (OR 2.110, 95% CI 1.317–3.380, *p* = 0.002) and baseline total bilirubin >21 μmol/L (OR 1.867, 95% CI 1.107–3.147, *p* = 0.019) were identified as risk factors for tigecycline-associated hypofibrinogenemia, while skin and soft tissue infection (OR 0.110, 95% CI 0.026–0.473, *p* = 0.003) was identified as a protective factor (Table 4).

## Discussion

Tigecycline has become one of the most powerful antibiotics for the treatment of on-label and off-label infections, including complicated intra-abdominal infections, complicated skin and skin-structure infections, community-acquired pneumonia, and hospital-acquired pneumonia. However, its recent widespread clinical application has highlighted the occurrence of coagulopathies, mainly characterized by hypofibrinogenemia, as an emerging adverse event. To avoid confounding factors such as region, comorbidities and disease severity, we therefore conducted a multicenter, retrospective, controlled study to assess the coagulopathy associated with tigecycline.

In this cohort study, we evaluated the effect of tigecycline on coagulation using imipenem-cilastatin as a comparator, given that both tigecycline and imipenem-cilastatin were the primary antibiotics for the treatment of GNB infections, especially MDR-GNB infections. However, there were still some differences in the demographic and clinical characteristics between two groups. We carried out a propensity score-matched analysis to correct for selection bias. Analyses of the original and propensity score-matched datasets both demonstrated that tigecycline was associated with reduced fibrinogen and prolonged aPTT and PT, with the most remarkable change in fibrinogen. At baseline balance, the incidence of hypofibrinogenemia in patients receiving tigecycline was >50% and tigecycline had an approximately 3-fold risk of developing hypofibrinogenemia compared to imipenem–cilastatin, which mean hypofibrinogenemia was probably caused by tigecycline. This was consistent with previously published incidences of hypofibrinogenemia of 56% and 50.5% in patients with tigecycline in two Chinese reports (Hu et al., 2020; Zhang et al., 2020), but differed from the results of Hakeam et al. and Campany-Herrero et al., who reported incidences of hypofibrinogenemia of only 5% and 19.4%, respectively (Hakeam et al., 2018; Campany-Herrero et al., 2020). This difference may be explained by differences in the study subjects, given the high incidence in China and the low incidence in other countries. However, it need to be validated in more studies in different regions and different ethnic groups.

In our study, patients treated with tigecycline developed hypofibrinogenemia after a median of 6.0 days and most of them (80.5%) had a fibrinogen nadir of 1–2 g/L. Hypofibrinogenemia led to an increased risk of bleeding, especially gastrointestinal and mucocutaneous hemorrhage. In addition, hypofibrinogenemia can even be life-threatening, Ciftciler et al. (2019) found that low level of fibrinogen was a risk factor for acute promyelocytic leukemia-induced early deaths. Therefore, once hypofibrinogenemia occurs, it is very important to take timely measures to reverse it. However, of the 44 patients who received fibrinogen concentrate during treatment, only 10 patients returned to normal levels of fibrinogen. Fibrinogen levels became normalized in patients with hypofibrinogenemia at a median of 4.0 days after withdrawal of tigecycline, with 46.3% receiving supplementation after withdrawal. By evaluating the commonly used supplemental therapies (vitamin K1, plasma and/or cryoprecipitate and fibrinogen concentrate), it was found that the use and type of supplemental therapy after withdrawal had no effect on the normalization of fibrinogen levels. Given that there was currently no known method to remarkably reverse hypofibrinogenemia during or after treatment, discontinuation of tigecycline is considered to be the preferable option when hypofibrinogenemia is detected.

A previous retrospective study of 127 patients suggested that tigecycline-induced hypofibrinogenemia was linked to intra-abdominal infection, fibrinogen levels at tigecycline initiation, maintenance dose and treatment duration (Hu et al., 2020). In another study, treatment duration >4 weeks, high-dose tigecycline and high protein C were identified as independent variables associated with fibrinogen reduction >1.7 g/L (Campany-Herrero et al., 2020). In the present study we came to the different results. Skin and soft tissue infection was identified as a protective factor for tigecycline-associated hypofibrinogenemia, although we are currently unable to speculate on a possible cause. Treatment duration ≥6 days was identified as a strong risk factor for tigecycline-associated hypofibrinogenemia. This cut-off value for treatment duration was much shorter than the previous study, but we believe that it is more appropriate, given that the half-life of fibrinogen is only 3–4 days. Contrary to the earlier studies, we found cumulative dose, rather than first dose and maintenance dose, to be a risk factor for tigecycline-associated hypofibrinogenemia. Thus, we believe that the potential risk of hypofibrinogenemia does not justify limiting the first or maintenance dose of tigecycline. In this study, baseline fibrinogen <4 g/L, baseline PT > 14 s and baseline total bilirubin >21 μmol/L were identified as risk factors for tigecycline-associated hypofibrinogenemia. This is the first time that a cut-off value has been introduced in the baseline coagulation parameter, which is very useful for the timely identification of at-risk patients. However, it is unclear why baseline PT but not aPTT was linked to the development of hypofibrinogenemia. Baseline total bilirubin >21 μmol/L as a newly identified risk factor may be related to increased concentrations of tigecycline induced by its limited biliary excretion.

Regarding concomitant therapy, a retrospective study using univariate analysis showed that renal failure promoted the occurrence of hypofibrinogenemia (Zhang et al., 2020). However, CRRT, but not a history of chronic kidney disease or elevated serum creatinine, was a risk factor for hypofibrinogenemia in this study. This association may be caused by the potential adsorption of fibrinogen to the CRRT membrane (Urbani et al., 2012), thus increasing the incidence of tigecycline-associated hypofibrinogenemia. Among the drugs commonly combined with tigecycline, cefoperazone–sulbactam, sodium valproate and asparaginase were reported to be associated with abnormal coagulation (Kumar et al., 2019; Wang et al., 2020; Vittayawacharin & Khuhapinant, 2021). In this study we did not observe an increased risk of hypofibrinogenemia with combination drugs.

The mechanism responsible for tigecycline-associated coagulopathy is unclear. The poor correlation among coagulation parameters suggests that the coagulopathy associated with tigecycline may involve multiple pathways and mechanisms. It has been suggested that inhibition of vitamin K-producing flora in the gut by tigecycline plays an important role in coagulation disorders (Sabanis et al., 2015). However, this cannot explain the reduction in plasma fibrinogen because vitamin K is not required for fibrinogen synthesis. The reduction in fibrinogen in general is likely to be due to its increased consumption and impaired synthesis. Two previous studies based on *in vitro* and clinical evidence, respectively, excluded the possibility that tigecycline’s effect on fibrinogen was related to its consumption (Leng et al., 2019; Brandtner et al., 2020). Fibrinogen is synthesized in the liver and Treml et al. (2021) attributed decreased fibrinogen levels to impaired liver synthesis. Moreover, a rapid loss of mitochondrial activity in hepatic cells was observed after the addition of tigecycline (Brandtner et al., 2020). Further laboratory studies are required to elucidate the mechanism by which tigecycline affects fibrinogen and other coagulation parameters.

This study had several limitations. First, it was a retrospective study and was thus susceptible to selection bias. Although propensity score-matched methods can reduce bias in estimates due to observed differences, there may still have been potential sources of biases from unobserved differences. Second, we could not exclude other factors that may affect the accuracy of the study, such as uncertainty in the timing and number of laboratory tests. Third, information on certain indicators was not available, such as Acute Physiology and Chronic Health Evaluation II, which is not routinely used to evaluate non-ICU patients. Fourth, many cases were excluded due to incomplete laboratory data (e.g., some non-critically ill patients were excluded from the study because no laboratory tests were performed on these patients during the administration), which may introduce selection bias.

## Conclusion

This study revealed that tigecycline is associated with a higher incidence of hypofibrinogenemia compared with imipenem–cilastatin. The characteristics identified in the development and recovery of tigecycline-associated hypofibrinogenemia could help us understand this adverse event. The risk factors, including treatment duration ≥6 days, baseline fibrinogen <4 g/L, cumulative dose ≥1,000 mg, receiving CRRT, baseline PT > 14 s and baseline total bilirubin >21 μmol/L, can offer useful early predictors to guide the management of infected patients receiving tigecycline. However, further large prospective studies are needed to validate and expand on these findings.

## Data Availability

The original contributions presented in the study are included in the article/Supplementary Material, further inquiries can be directed to the corresponding author.
